# Three genes controlling streptomycin susceptibility in *Agrobacterium fabrum*

**DOI:** 10.1128/jb.00165-23

**Published:** 2023-09-11

**Authors:** Robyn E. Howarth, Curtis M. Pattillo, Joel S. Griffitts, Diana G. Calvopina-Chavez

**Affiliations:** 1 Department of Microbiology and Molecular Biology, Brigham Young University, Provo, Utah, USA; Philipps-Universitat Marburg Fachbereich Biologie, Marburg, Germany

**Keywords:** streptomycin resistance, *Agrobacterium fabrum*, *rpsL*, *rsmG*, *gidB*, *strB*

## Abstract

Streptomycin (Sm) is a commonly used antibiotic for its efficacy against diverse bacteria. The plant pathogen *Agrobacterium fabrum* is a model for studying pathogenesis and interkingdom gene transfer. Streptomycin-resistant variants of *A. fabrum* are commonly employed in genetic analyses, yet mechanisms of resistance and susceptibility to streptomycin in this organism have not previously been investigated. We observe that resistance to a high concentration of streptomycin arises at high frequency in *A. fabrum*, and we attribute this trait to the presence of a chromosomal gene (*strB*) encoding a putative aminoglycoside phosphotransferase. We show how *strB*, along with *rpsL* (encoding ribosomal protein S12) and *rsmG* (encoding a 16S rRNA methyltransferase), modulates streptomycin sensitivity in *A. fabrum*.

**IMPORTANCE:**

The plant pathogen *Agrobacterium fabrum* is a widely used model bacterium for studying biofilms, bacterial motility, pathogenesis, and gene transfer from bacteria to plants. Streptomycin (Sm) is an aminoglycoside antibiotic known for its broad efficacy against gram-negative bacteria. *A. fabrum* exhibits endogenous resistance to somewhat high levels of streptomycin, but the mechanism underlying this resistance has not been elucidated. Here, we demonstrate that this resistance is caused by a chromosomally encoded streptomycin-inactivating enzyme, StrB, that has not been previously characterized in *A. fabrum*. Furthermore, we show how the genes *rsmG*, *rpsL*, and *strB* jointly modulate streptomycin susceptibility in *A. fabrum*.

## INTRODUCTION

**Fig 1:**
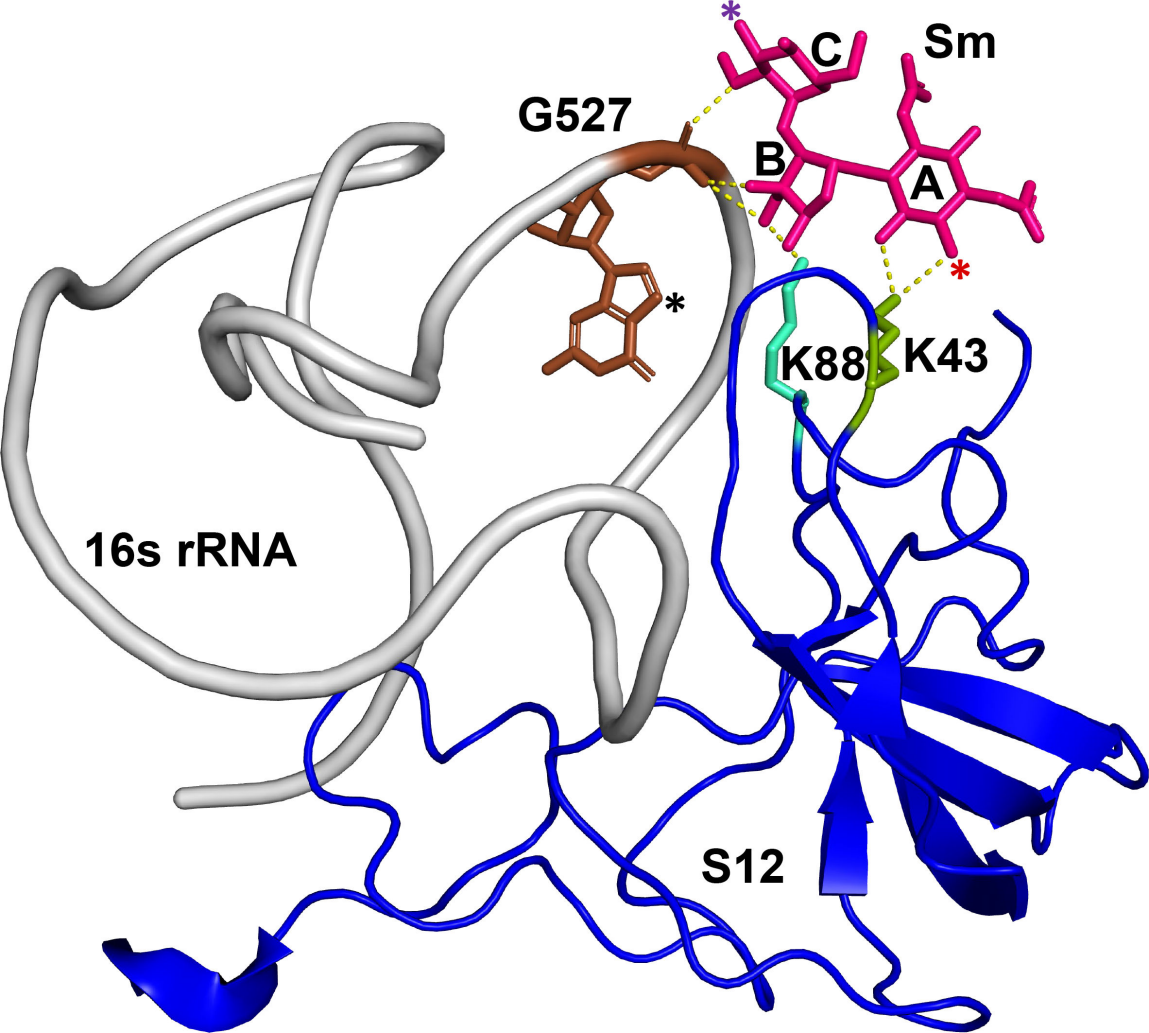
Cartoon representation of a portion of the 30S subunit from *Thermus thermophilus* that shows key interactions with Sm (adapted from PDB ID: 1FJG) (2). Sm is shown in pink sticks. The letters A, B, and C show the streptidine ring, l-streptose ring, and *N*-methyl-l-glucosamine ring, respectively. The red asterisk shows the 6-hydroxyl that gets phosphorylated by APH(6), while the purple asterisk indicates the 3′′-hydroxyl that gets phosphorylated by APH(3′′). Helix 18 of the 16s rRNA is shown in gray with guanosine 527 highlighted in brown. The black asterisk shows the nitrogen atom that is methylated by RsmG. Ribosomal protein S12 is shown in blue with key residues K88 and K43 shown in cyan and green, respectively.

Streptomycin (Sm) inhibits the fidelity of the prokaryotic ribosome by stabilizing a conformational state of the 16S rRNA that results in codon-anticodon mismatches during translation (1). Sm binds the ribosome at an interface between several 16S helices, including helix 18, and the ribosomal protein S12 (Fig. 1) (2, 3). Sm resistance often results from mutations in *rpsL*, *rsmG* (also known as *gidB*), and *rrs* which, respectively, encode ribosomal protein S12, *S*-adenosylmethionine (SAM)-dependent 16S rRNA methyltransferase (RsmG), and 16S rRNA.

In diverse bacteria, high-level streptomycin resistance can be caused by point mutations in the S12-encoding *rpsL* gene (4, 5). S12 is located at the interface of the large and small ribosomal subunits, where it interacts with the EF-Tu-bound tRNA acceptor arm and functions as a control element for translocation of the mRNA:tRNA complex (6 - 8). In *Escherichia coli*, spontaneous mutations in the *rpsL* gene that result in a single amino acid change (K42R or K87R) confer high levels of Sm resistance (9, 10). These mutations also occur in Sm-resistant strains of *Mycobacterium tuberculosis* and *Streptomyces coelicolor* (11 - 13).

RsmG is a member of a large family of SAM-dependent methyltransferases functioning in cell division and chromosome replication. In many bacteria, such as *E. coli* and *Bacillus subtilis*, RsmG has been shown to be responsible for N7 methylation of the 16S rRNA at position G527 located on the highly conserved helix 18 (Fig. 1) (14, 15). Sm has been shown to interact with the phosphate backbone of G527 (3, 16). In *S. coelicolor, M. tuberculosis*, *B. subtilis*, and *Thermus thermophilus*, loss of *rsmG* results in low-level Sm resistance likely due to the loss of this key methylation event occurring near the Sm binding pocket (14, 15, 17 - 19).

Alterations in the 16S sequence are generally not associated with Sm resistance because most bacteria possess many redundant copies of the 16S-encoding gene (*rrs*), making any single *rrs* mutation recessive. However, mutations in the *rrs* gene that confer Sm resistance can be found by genetically modifying bacteria to carry a single functional copy of *rrs* and selecting for Sm-resistant mutants. For example, in *M. smegmatis*, mutations in the *rrs* gene were selected by altering the number of *rrs* alleles in the bacterial genome. Most of the mutations mapped to the highly conserved 530 loop region of the 16S rRNA, specifically the mutation 524G>C which has been thought to be essential for ribosome function (20).

Sm resistance may also be conferred by Sm-inactivating enzymes. The *strA-strB* resistance cassette has been characterized in taxonomically diverse Gram-negative bacteria. StrA is an aminoglycoside-3″-phosphotransferase [APH(3″)] that catalyzes the addition of a phosphate group from ATP to the 3″ hydroxyl of the *N*-methyl-l-glucosamine ring of Sm (21). StrB is an aminoglycoside-6-phosphotransferase [APH(6)], which phosphorylates the 6-hydroxyl group of the streptidine ring of Sm yielding streptomycin 6-phosphate and ADP (21, 22). In both cases, the resulting streptomycin phosphate (streptomycin 3″’-phosphate and streptomycin 6-phosphate) is inactivated and can no longer bind to the ribosome (23, 24). The pair of enzymes, StrA-StrB, is thought to work in concert to inactivate Sm, with the loss of either gene being associated with the loss of strong resistance (25 - 28).

The plant pathogen, *Agrobacterium fabrum* (formerly *A. tumefaciens*), has become an important model for studying interkingdom gene transfer, cell polarity, and motility (29, 30). The genome of *A. fabrum* strain C58 is composed of a 2,841,581 bp circular chromosome; a 2,075,600 bp linear chromosome; a 542,869 bp AT plasmid; and the 214,233 bp Ti virulence plasmid (31). *A. fabrum* lives in diverse plant-associated environments such as vegetation, rhizosphere, and soil; therefore, it is constantly challenged by multiple stressors which include plant defenses, microbial competition, and antibiotics used in plant agriculture such as streptomycin (32). Here, using a plasmid-free derivative strain (UBAPF2), we report that *A. fabrum* has moderate Sm resistance due to an unusual chromosomal copy of *strB* without an accompanying *strA* companion gene. In this context, we show how Sm susceptibility is controlled in *A. fabrum* by the *strB*, *rsmG*, and *rpsL* gene networks.

## RESULTS

### Frequency and mechanism of Sm resistance in *A. fabrum* vary by Sm concentration

A plasmid-free derivative of *A. fabrum* C58 (UBAPF2) (33) was found to give rise to surprisingly large numbers of Sm-resistant colonies when selected at 200 µg/mL Sm, with an average frequency of 7.1 × 10^−5^ ± 2.3 × 10^−5^ (SD; *n* = 10). However, at 800 µg/mL, colonies emerged over 100 times less frequently, with an average frequency of 4.3 × 10^−7^ ± 2.1 × 10^−7^ (SD; *n* = 10). Each culture in these analyses was derived from an independent colony in order to account for fluctuation in the data. We sequenced *rpsL* for several Sm^200^- and Sm^800^-resistant derivatives and found sequence changes only in the Sm^800^ group (with a major allele being K43R), suggesting that the mechanism of resistance for Sm^200^ derivatives is not mediated by *rpsL*.

**Fig 2:**
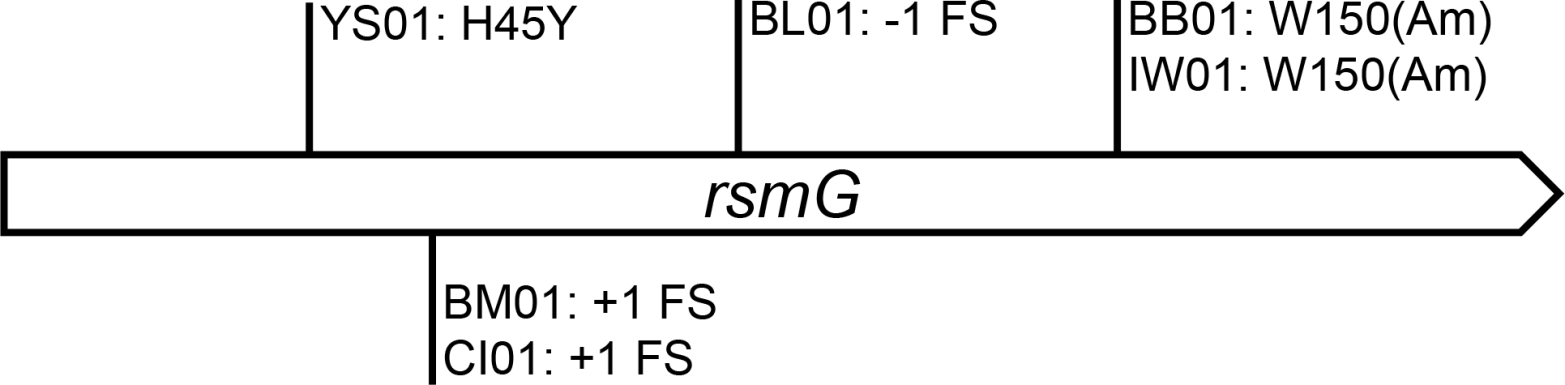
Spontaneous mutant alleles of *rsmG* associated with Sm^200^ resistance in *A. fabrum*. Six independently derived isolates (YS01, BM01, CI01, BL01, BB01, and IW01) selected on Sm were whole-genome sequenced, and each exhibited a mutation in *rsmG* (FS, frameshift; Am, premature amber stop codon).

To determine the genetic basis of resistance in Sm^200^ derivatives, whole-genome resequencing was carried out on six independent isolates. In each of the isolates, a mutation was found in *rsmG* (*ATU2830*, also known as *gidB*), and these are depicted on the map in Fig. 2. These *rsmG* alleles are mostly predicted to be associated with loss of function due to frameshift or nonsense mutations. Aside from these six mutations in *rsmG*, only two additional sequence deviations from the reference genome were identified across the six resequenced strains: one intergenic substitution in strain YS01 and one missense mutation (Gly to Ala) in the riboflavin biosynthesis *ribB* gene in strain BB01. From this, we conclude that Sm^200^ resistance in these UBAPF2 derivatives was brought about by the observed changes in *rsmG*. To confirm this, we introduced a normal copy of *rsmG* on a plasmid into one of our *rsmG* frameshift mutants (D337) and found that this complementation plasmid reduced the Sm sensitivity back to the wild-type level (Fig. S1).

### *strB* provides background Sm resistance in *A. fabrum*

**Fig 3:**
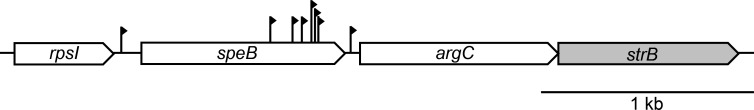
Tn5-110 transposon insertions giving rise to Sm^200^ resistance in *A. fabrum*. Mapped insertion sites are indicated by vertical lines. Direction of transcription from the strong P_trp_ promoter on the transposon is indicated by filled arrowheads. The *strB* gene suspected of being required for this resistance is highlighted in gray.

**Fig 4:**
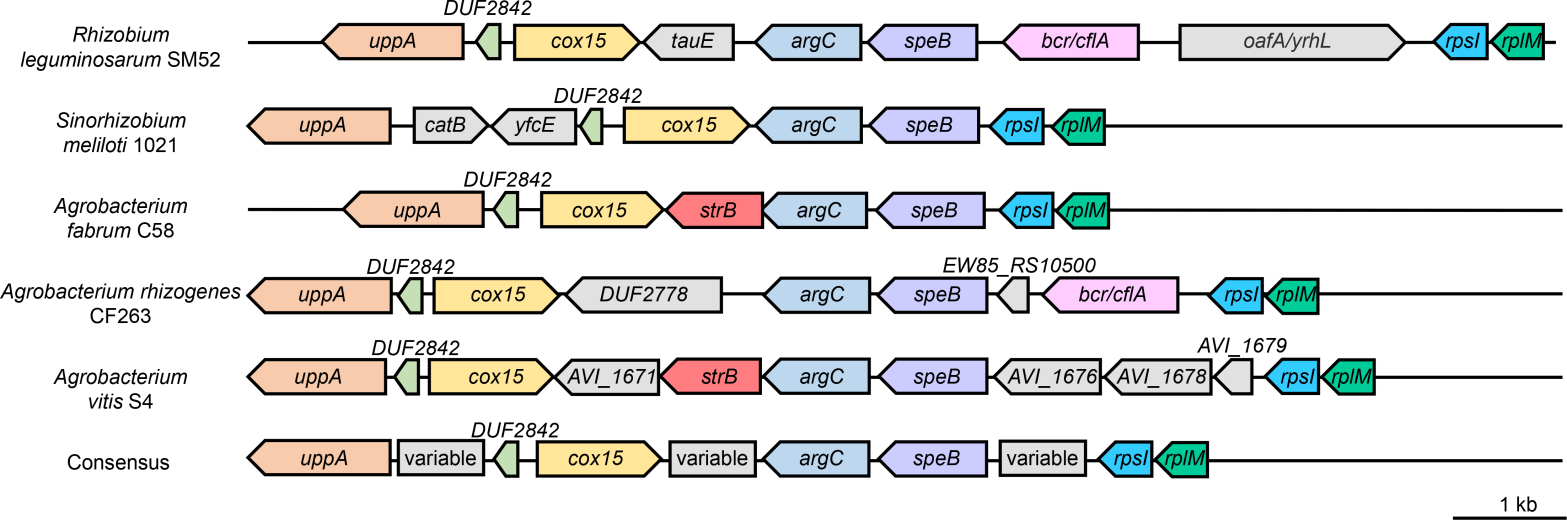
Interspecies comparison of the *strB* genomic region. Conserved genes across species are depicted in the same colors, while variable genes are shown in gray. The *strB* gene is depicted in red.

For many Gram-negative, Sm-sensitive bacteria, Sm^200^ is considered a high dose. In *E. coli* K12, for example, we observe resistance to Sm^200^ to occur at a frequency of less than 1 × 10^−9^, and the mechanism is uniformly *rpsL*-mediated (Table S1; online supplementary file 1). Mutations in *rsmG* are generally associated with low-level Sm resistance (14, 15, 17). This suggests that the parental UBAPF2 strain possesses significant background resistance. Investigating this further, we found the minimal inhibitory concentration (MIC) of Sm to be around 128 µg/mL for UBAPF2. We hypothesized that this native-level resistance is caused by an endogenous, dominantly acting gene. We reasoned that random chromosomal insertion of a strong promoter could help us identify this factor by screening for elevated Sm resistance. The Tn5-110 transposon carries the outwardly oriented P_trp_ promoter from *Salmonella* (34) that has successfully given overexpression phenotypes in *Sinorhizobium meliloti*. We conjugated the Tn5-110 delivery plasmid into UBAPF2 and selected for growth on Sm^200^ plates (additionally containing neomycin to select for transposon insertion). Fifteen colonies from this selection were evaluated for transposon insertion location. In seven of these, insertions were distributed around the genome with no clear pattern (Table S2; online supplementary file 1); in the other eight, the insertions occurred in varying positions within a small genomic interval, shown in Fig. 3. These eight insertions all position the P_trp_ promoter in the same orientation, reading into a pair of likely co-transcribed genes: ATU1244 and ATU1243. We presume these two genes are co-transcribed because (i) they are transcribed in the same orientation, (ii) the last 4 bp of the ATU1244 coding sequence overlap with the ATU1243 coding sequence, and (iii) by using ARNold (35, 36) and RhoTermPredict (37) tools, we could not detect any transcription terminators within 150 bp downstream of ATU1244. ATU1244 (*argC*) encodes an *N*-acetyl-gamma-glutamyl-phosphate reductase enzyme predicted to be involved in the biosynthesis of arginine and ornithine. Downstream, ATU1243 (*strB*) encodes an StrB family phosphotransferase, possibly involved in modification of streptomycin or similar aminoglycoside antibiotics. This gene has not been previously associated with Sm resistance in *A. fabrum*. Considering that *strB* genes are usually linked to *strA* partner genes (22, 26, 38), we sought to identify potential *strA* homologs in *A. fabrum*. In a BlastP search against the *A. fabrum* C58 genome (which encompasses the circular and linear chromosomes as well as the AT and Ti plasmids) using the canonical StrA/StrB protein sequences encoded by *E. coli* plasmid RSF1010 (25, 27, 39), we identified the *A. fabrum strB* gene reported above, but no homolog for *strA*. The *A. fabrum strB* gene is encoded on the circular chromosome and resides in a genomic region that is generally conserved across many species in the Rhizobiaceae family. For example, the *speB-argC* gene pair found upstream of *strB* is well conserved in this family, as well as nearby ribosomal protein genes *rpsI and rplM*. However, interspecies comparison of this genomic region in *Rhizobium leguminosarum* SM52, *Sinorhizobium meliloti* 1021, *A. fabrum* C58*, A. rhizogenes* CF263, and *A. vitis* S4 shows that it is generally conserved but punctuated by certain species-specific genes (Fig. 4). One of these variable genes is *strB*, which is found in some *Agrobacterium* species but absent in the other Rhizobiaceae genera that we evaluated. In *A. fabrum* C58, *strB* does not appear to bear any features relating to mobile genetic elements (tRNA, integrase or transposase genes), and *strB* codon usage is consistent with the rest of the genome. This suggests that the introduction of *strB* into agrobacteria is not very recent.

**Fig 5:**
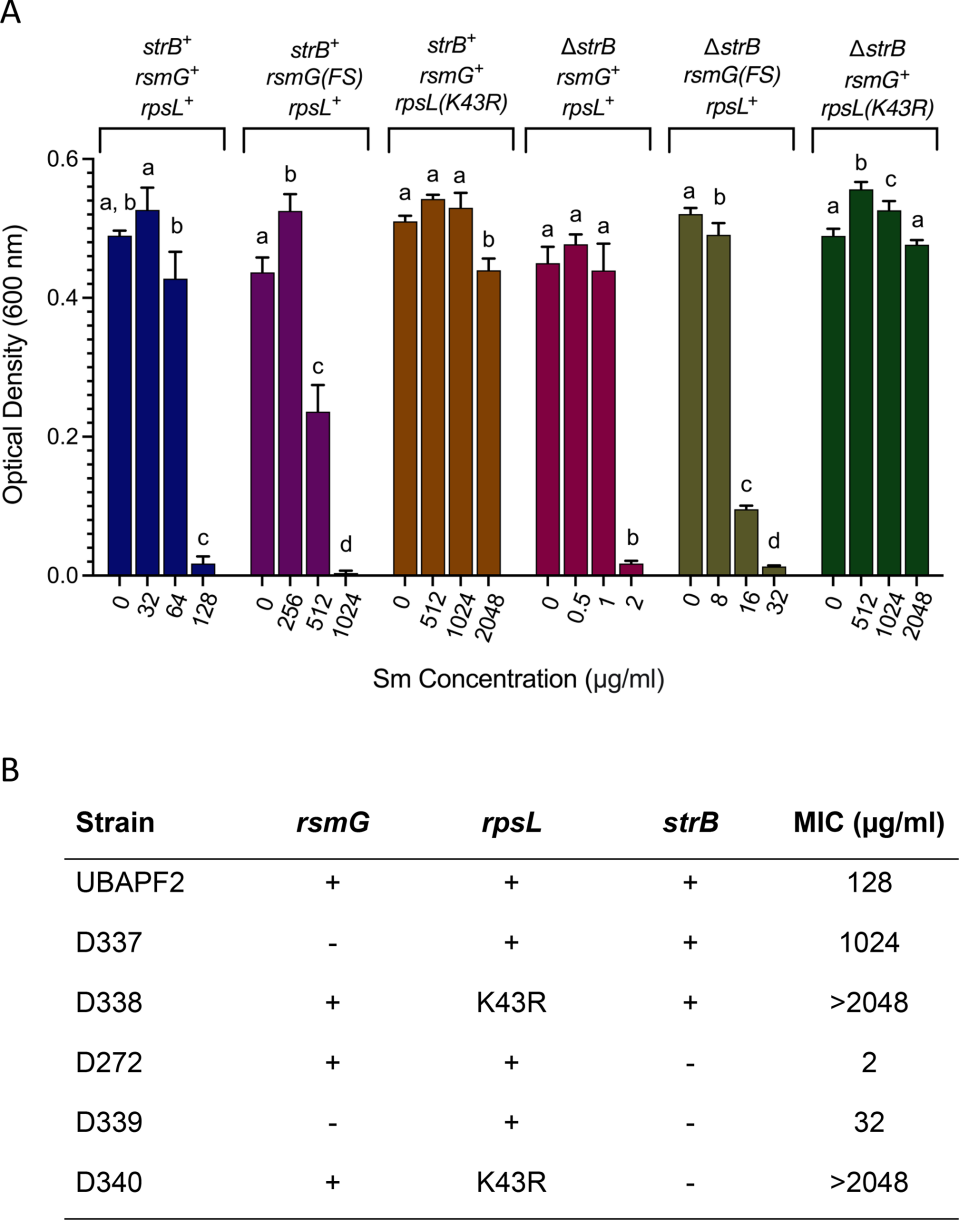
Sm dose responses for six *A. fabrum* genotypes tested. (**A**) Optical density measurements were taken 20 h after inoculation of 200 µL cultures in 96-well plates. Genotype descriptions are given above each set of growth values. Error bars represent the standard deviation from the mean (*n* = 3). Different letters denote statistically significant differences (*P* < 0.05) according to a Tukey multiple comparison test. (**B**) Another representation of data is shown in (**A**), indicating MIC values.

We reasoned that deletion of *strB* would significantly reduce the Sm resistance observed for our parental strain. To test this, *strB* was removed using allele exchange, leaving only the first and last 10 codons of the gene intact. The Δ*strB* deletion strain was found to have over 60-fold greater sensitivity to Sm, with an MIC of 2 µg/mL (see Fig. 5). When complemented with a plasmid-borne copy of *strB*, the Δ*strB* strain rebounded to an MIC of 2,048 µg/mL (Fig. S1), a value much higher than the wild type. In the Δ*strB* genetic background, we found that Sm-resistant colonies arise at low frequency (approximately 5 × 10^−7^) on both 200 and 800 µg/mL Sm, suggesting that *rpsL*-mediated resistance is the predominant mechanism under both conditions. Indeed, all colonies analyzed from these selections (4/4 for 200 µg/mL and 4/4 for 800 µg/mL) harbored *rpsL* mutations. Six of these had the K43R allele, and two of the Sm^200^-resistant mutants had the K88R allele (Fig. 1).

**Fig 6:**
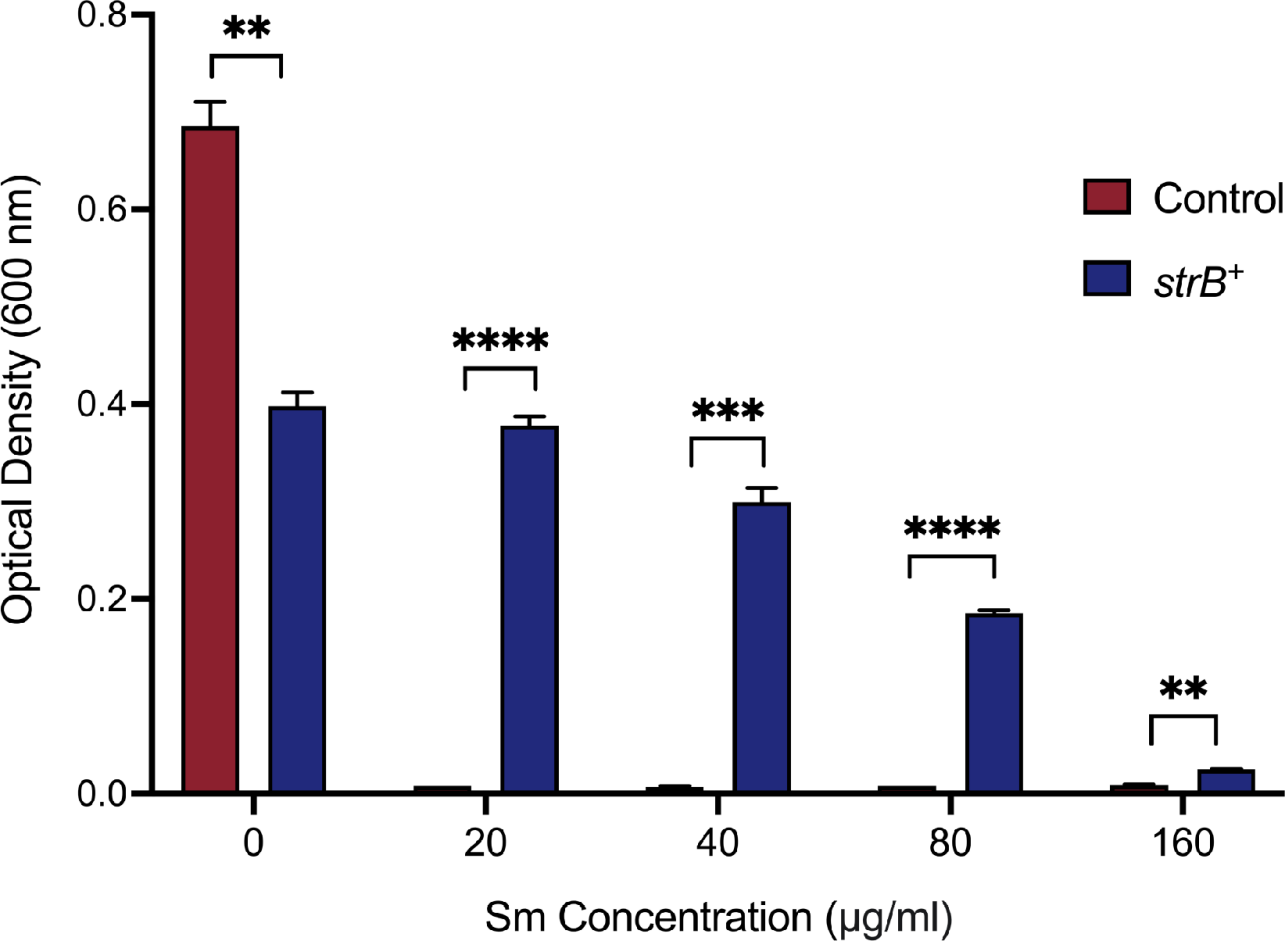
Dose responses for two strains of *E. coli* in the presence of Sm. The *strB*^+^ strain (blue) contains a plasmid that constitutively expresses *A. fabrum strB,* while the control strain (red) contains the empty parent vector. Error bars show the standard deviation from the mean (*n* = 3). Significant differences (****P < 0.000001, ***P < 0.000005, **P < 0.00007) are indicated by asterisks according to parametric *t*-tests carried out with the Benjamini, Krieger, and Yekutieli method.

To test the sufficiency of *A. fabrum strB* to confer Sm resistance in *E. coli*, it was ligated into a small constitutive expression plasmid and tested for its ability to provide Sm resistance to *E. coli* strain DH5α. As shown in Fig. 6, this plasmid allowed the growth of *E. coli* up to 160 µg/mL Sm, whereas the vector-only control strain was unable to grow at all non-zero doses tested. The strain expressing *strB* exhibited a significant growth defect in the absence of Sm, likely a result of a metabolic cost from the constitutive expression of this resistance gene.

### *rpsL*, *rsmG*, and *strB* constitute a three-gene network modulating Sm resistance in *A. fabrum*

The results outlined thus far point to a model in which three different *A. fabrum* genes influence Sm sensitivity: *strB* provides a modest level of resistance by inactivation of the antibiotic, *rsmG* loss of function can boost resistance by subtly altering the Sm binding site on the ribosome without greatly affecting strain fitness, and very rare and specific mutations in the essential *rpsL* gene can confer greatly elevated resistance due to binding site alteration. This model predicts that the impact of *rsmG* loss of function is strongly modulated by the presence or absence of *strB*, but that Sm resistance-associated *rpsL* mutations provide very strong resistance whether *strB* is present or not. Six genotypes were constructed to test this: (i) *strB^+^ rsmG^+^ rpsL^+^*, (ii) *strB^+^ smG(FS) rpsL^+^,* (iii) *strB^+^ rsmG^+^ rpsL(K43R*), (iv) Δ*strB rsmG^+^ psL^+^*, (v) Δ*strB rsmG(FS) rpsL^+^,* and (vi) Δ*strB rsmG^+^psL(K43R*). Sm dose-response data for these six strains are given in Fig. 5. We see from this that tolerance to Sm across all six strains was consistent with our model. The *rpsL(K43R*) allele confers extremely high resistance, whether or not *strB* is intact; *rsmG* loss of function modestly enhances resistance in the presence or absence of *strB*. Remarkably, *rsmG* loss of function increases resistance by a similar factor (~10-fold) in the presence or absence of *strB*, indicating that the influence of each gene on resistance is independent and additive.

## DISCUSSION

In this study, three genes were found to have an effect on *A. fabrum* resistance to Sm. A chromosomal *strB* homolog provides moderate resistance which can be enhanced by mutations in either *rsmG* or *rpsL*, the former yielding resistance at higher frequency and the latter conferring resistance to higher Sm doses. The marked difference in frequency of resistance brought about by changes in *rsmG* compared to *rpsL* may be explained by the essentiality of *rpsL* function for cell viability, and so only special alleles of *rpsL* can support both viability and resistance (9). The *rsmG* gene, on the other hand, does not appear to be essential for viability though deficiency in this gene is associated with only modest resistance to Sm (14, 15, 17). K43R and K88R missense mutations in *rpsL* have been correlated with high-level Sm resistance in several species of bacteria including *Yersinia pestis* (40) and *M. tuberculosis* (41), indicating the conserved molecular-level conservation of Sm binding to this region of the ribosome, and the narrow spectrum of allelic variants of rpsL that can support both viability and Sm resistance.

The *strB* gene in *A. fabrum* is not accompanied by a homolog of *strA*. Orphan *strA*-only or *strB*-only loci are rarely encountered in bacteria, and this report is the only one to date in which such a locus has been functionally characterized. Where *strA-strB*-encoded aminoglycoside phosphotransferases have been best characterized (RSF1010 from *E. coli*, pPSR1 from *Pseudomonas syringae,* and transposon Tn*5393* from *Erwinia amylovora*), the *strA* and *strB* genes are co-transcribed in a single operon. The logical assumption has been that simultaneous 3″-phosphorylation (*strA*) and 6-phosphorylation (*strB*) of the Sm molecule provide more robust resistance than either modification alone. This was shown to be the case for the Tn*5393* locus, where removal of *strA* (retaining *strB*) decreased the MIC by more than 20-fold and removal of *strB* (retaining *strA*) decreased MIC by more than 5-fold (22). In another study, overexpression in *E. coli* of *strB* derived from pPSR1 conferred Sm resistance with an MIC of 200 µg/mL (23), which is similar to the resistance conferred to *E. coli* by the *A. fabrum* variant in this study (MIC of 160 µg/mL). However, over-expression of the *A. fabrum* variant in *A. fabrum* supports a much higher level of resistance (MIC of 2,048 µg/mL). This observation raises the possibility that the relative importance of 3″-phosphorylation (forming Sm 3″-phosphate) compared to 6-phosphorylation (forming Sm 6-phosphate) may vary according to the bacterial target. For example, simultaneous modifications may be required for robust resistance in *E. coli*, while 6-phosphorylation is sufficient for resistance in *A. fabrum*. This implies that the ribosomal binding pockets in the two organisms may be slightly different, with the *A. fabrum* pocket being less compatible with Sm-6-phosphate binding than the analogous pocket in *E. coli*.

Certain organisms encode redundant enzymes that inactive Sm. For instance, *Streptomyces griseus*, a soil-dwelling Sm-producing bacterium, harbors a gene for the enzyme APH(6)-Ia, which protects this organism against the toxic effects of its own antibiotic. While APH(6)-Ia catalytic activity is enough to inactive Sm, *S. griseus* contains a second Sm inactivating enzyme APH(3″)-Ia, which is located outside the Sm biosynthetic gene cluster (40 - 42). One explanation for expressing redundant Sm inactivating enzymes is that APH(6) enzymes are considerably less efficient at inactivating Sm through phosphorylation compared to other phosphotransferases that inactive similar aminoglycosides (23, 42 - 44), so a second phosphorylating enzyme may be required for effective inactivation of Sm. Another potential reason for carrying redundant Sm-inactivating enzymes is that some aminoglycoside phosphotransferases can provide a broad-spectrum resistance to other aminoglycoside antibiotics. For example, the bifunctional enzyme AAC(6′)-Ie-APH(2″)-Ia contains acetyltransferase and phosphotransferase functional domains and provides the host with resistance to a wide range of aminoglycoside antibiotics (45). However, when APH(3″) from *S. griseus* was functionally characterized, it did not detectably phosphorylate other aminoglycoside antibiotics such as neomycin or kanamycin which shows high substrate specificity to Sm (21).

In the several decades since *strA-strB* gene pairs were initially discovered, the evolutionary explanation for their coexpression has not been satisfactorily resolved. Given that these genes are often encoded on invasive DNA elements (plasmids and transposons), expression of both may be a form of bet-hedging to ensure that diverse hosts will be protected, where some hosts are more protected by the *strA*-dependent modification and others more protected by the *strB*-dependent modification. A multispecies analytical system would make this notion somewhat straightforward to test. Our observations relating to *strB* in *A. fabrum* are notable in two respects: first is that it is not associated with an *strA* homolog, and second is that it is located on the chromosome rather than on a plasmid. This work expands the list of aminoglycosides phosphotransferases that have been studied to date and illustrates the distribution of Sm resistance genes present in soil-dwelling bacteria, which carries agricultural significance considering that Sm is one of the most commonly used antibiotics in plant agriculture for bacterial disease control.

## MATERIALS AND METHODS

### Bacterial strains and growth conditions

*A. fabrum* and *E. coli* strains were grown in Luria Broth (LB) containing (per liter) 10 g tryptone, 5 g yeast extract, 5 g NaCl, and 1 mL of 2 N NaOH, with 12 g of agar added to solidify when appropriate. *A. fabrum* was grown at 30°C for 2 days, while *E. coli* was grown overnight at 37°C. Where appropriate, antibiotics were used as follows: streptomycin (Sm), typically 200 or 800 µg/mL; chloramphenicol (Cm), 30 µg/mL; kanamycin (Km), 30 µg/mL; neomycin (Nm), 100 µg/mL; and rifampicin (Rf), 100 µg/mL. When needed, LB was supplemented with 100 µg/mL of 5-bromo-4-chloro-3-indoxyl-beta-d-glucuronide cyclohexylammonium salt (X-Gluc) and 1% sucrose. All strains and plasmids used in this study are given in Tables S3 and S4 (online supplementary file 1), respectively. Primer sequences are given in Table S5 (online supplementary file 1). Relevant plasmid sequences are given in Supplemental Materials.

### Calculating the frequency of spontaneous mutations

To evaluate the tendency of our starting strain UBAPF2 to mutate to Sm resistance, 10 independent colonies were grown to saturation in separate liquid cultures. From these, cells were plated on Sm^800^ (800 µg/mL), Sm^200^ (200 µg/mL) or no-Sm LB plates. From colony counts, mean frequencies (Sm^R^/Total) and standard deviation values were established.

### Selection of streptomycin-resistant mutants and *rpsL* Sanger sequencing

Six independent Sm^200^-resistant *A. fabrum* colonies were established as strains BB01, BL01, BM01, CI01, IW01, and YS01. The *rpsL* gene was amplified from each. PCR was carried out under standard conditions using Taq polymerase and primers 2,235 and 2,236. Lysed cells, used as template for PCR, were prepared by suspending cells in 200 µL of PCR lysis buffer (5 mM Tris pH 8.0, 2 mM EDTA, 0.5% Triton X-100) and heating to 95°C for 5 min with intermittent vortexing. PCR products were purified using the ZR Plasmid Miniprep-Classic Kit (Zymo Research), followed by Sanger sequencing using either primer 2,235 or 2,236.

### Whole-genome sequencing

Whole-genome sequencing was performed for the six Sm-resistant strains listed above, as well as the wild-type parent strain. Genomic DNA samples were produced with a final concentration greater than 20 ng/µL, using Proteinase K-mediated lysis followed by column purification using the DNeasy PowerLyzer Microbial Kit (Qiagen). These seven samples were sent to Microbial Genome Sequencing Center (MiGS) for Illumina sequencing to return 200 Mbp of data per strain. Resulting FASTQ files were inputted with the GenBank file for the C58 strain of *A. fabrum* into the command-line tool, *breseq*, using Windows Subsystem for Linux and R (46). The *breseq* output allowed for the discernment of sequence variants compared to the reference. Whole-genome raw sequence reads for all seven strains are available on NCBI under the BioProject accession number PRJNA993692 (https://www.ncbi.nlm.nih.gov/sra/PRJNA993692).

### Transposon mutagenesis and determination of insertion sites

A large-scale transposon mutagenesis was performed on *A. fabrum* using pJG110, the delivery plasmid for transposon Tn5-110 (34). A triparental mating was carried out to mobilize the transposon delivery plasmid into *A. fabrum*. This was done by combining the wild-type *A. fabrum* strain (UBAPF2; recipient), the donor strain (DH5α-pJG110), and the helper strain (B001) into a mixed suspension; plating mixed cells onto plain LB; and incubating at 30°C for 24 h. Resulting lawns were resuspended, plated onto LB-agar containing Sm (200 µg/mL) and Nm, and incubated at 30°C to select for transposants with Sm resistance. Twenty-four medium and large colonies were analyzed by arbitrary-PCR to determine transposon insertion sites. Bacterial template DNA for arbitrary-PCR was prepared by cell lysis as described above. Arbitrary-PCR was carried out as described by Calvopina-Chavez et al. (47), except primers 2,133 and 2,135 were used for the first-round PCR, and primers 2,134 and 2,137 were used for the second-round PCR. DNA products were purified as described above and Sanger sequenced using primer 2,134.

### Construction of the Δ*strB* strain D272

Allelic exchange plasmid pJG1108 (47), containing the *gus* and *sacB* genes, was used for *strB* deletion in UBAPF2. Primers oDC103 and oDC104 were designed to amplify the *strB* left homology region, and oDC105 and oDC106 were designed to amplify the right homology region. The two fragments were amplified by PCR using the high-fidelity Q5 polymerase, and they were inserted into XbaI/SalI-digested pJG1108 in a three-fragment ligation. Cloned inserts were then amplified and sequence-verified using primers CD49 and CD50. The resulting *strB* knock-out plasmid (pJG1197) was conjugated into *A. fabrum* UBAPF2 via triparental mating as described above for transposon mutagenesis though, in this case, single cross-over transconjugants were selected on Rf and Nm. Subsequent selection for plasmid eviction was carried out on LB containing X-Gluc and Sucrose. For several resultant white colonies, the deletion of *strB* was evaluated by colony PCR using Taq polymerase and primers oDC107 and oDC108. Products were then Sanger sequenced to confirm the deletion.

### Creating a plasmid for constitutive expression of *strB*

Parent plasmid pJG1226 consists of a p15A origin and a Cm resistance gene expressed from a constitutive P_trc_ promoter (pJG1226; sequence is given in Supplemental Materials). A segment with these elements was amplified from pJG1226 with primers oDC196 and oDC197 and digested with XbaI and HindIII. The *strB* gene was amplified from UBAPF2 genomic DNA with primers oDC198 and oDC199 and also digested with XbaI and HindIII. Ligation of the two fragments (pDC76) places *strB* immediately downstream of the *cat* (Cm^R^) gene such that the two are co-transcribed.

### Testing *strB*-dependent Sm resistance in *E. coli*

*E. coli* strain DH5α harboring either pDC76 or pJG1226 (vector-only) was grown in 5 mL of LB + Cm at 37°C overnight. Five microliters of overnight culture was added to 5 mL of LB + Cm containing 0, 20, 40, 80, or 160 µg/mL Sm in triplicates and allowed to grow for 6 h at 37°C. Optical density of each culture was measured at a wavelength of 600 nm, after which data were plotted. Unpaired parametric *t*-tests were carried out with the Benjamini, Krieger, and Yekutieli method (48) to determine statistically significant differences.

### Construction of allelic combinations of *strB*, *rsmG,* and *rpsL*

To learn how *strB*, *rsmG*, and *rpsL* interact to modulate Sm resistance in *A. fabrum*, six strains with different allelic combinations of these three genes were assessed: UBAPF2 (*strB^+^ rsmG^+^ rpsL^+^*), D337 [*strB^+^ smG(FS) rpsL^+^*], D338 [*strB^+^ rsmG^+^ rpsL(K43R*)], D272 (Δ*strB rsmG^+^ psL^+^*), D339 [Δ*strB rsmG(FS) rpsL^+^*], D340 [Δ*strB rsmG^+^ +psL(K43R*)]. The *rsmG(FS*) and *rpsL(K43R*) alleles arise spontaneously with sufficient frequency that they could be introduced by selection on Sm followed by PCR and sequence verification. The *rsmG(FS*) allele is a + 1 frameshift identical to the allele found in strains BM01 and CI01 (in a homopolymeric run around nt 177 of the *rsmG* coding sequence).

### Determining minimal inhibitory concentrations

MICs were determined by growing each strain in triplicate in a 96-well plate containing LB + Sm at appropriate concentrations. The six strains described in the previous paragraph were tested in three concentrations of Sm and a no-Sm control. Specific concentrations are given in Fig. 5. For these tests, each well contained 190 µL of LB and was inoculated with 10 µL of a 10^−1^ dilution of saturated overnight culture. For each strain tested, Sm concentrations were chosen so that the MIC could be discerned. 96-well plates were shaken for 20 h at 30°C, and culture densities were assessed by OD measurement at 600 nm. For each strain grown under the four conditions, a one-way ANOVA was carried out using Tukey’s multiple comparisons test.

## Data Availability

All relevant data are within the paper and its supplemental material.

## SUPPLEMENTAL MATERIAL

Fig. S1 jb.00165-23-s0001.docx Complementation with plasmid-borne *rsmG* and *strB* genes in *A. fabrum*.

Supplemental material jb.00165-23-s0002.docx Tables S1 to S5 and plasmid sequences.

ASM does not own the copyrights to Supplemental Material that may be linked to, or accessed through, an article. The authors have granted ASM a non-exclusive, world-wide license to publish the Supplemental Material files. Please contact the corresponding author directly for reuse.
