# Supplementary material for: Three genes controlling streptomycin susceptibility in Agrobacterium fabrum
Source: J Bacteriol. 2023 Sep 11;205(9):e00165-23. doi: 10.1128/jb.00165-23 (PMC10521367; doi:10.1128/jb.00165-23)
Supplement: Fig. S1 — Complementation with plasmid-borne rsmG and strB genes in A. fabrum. [file jb.00165-23-s0001.docx]

**
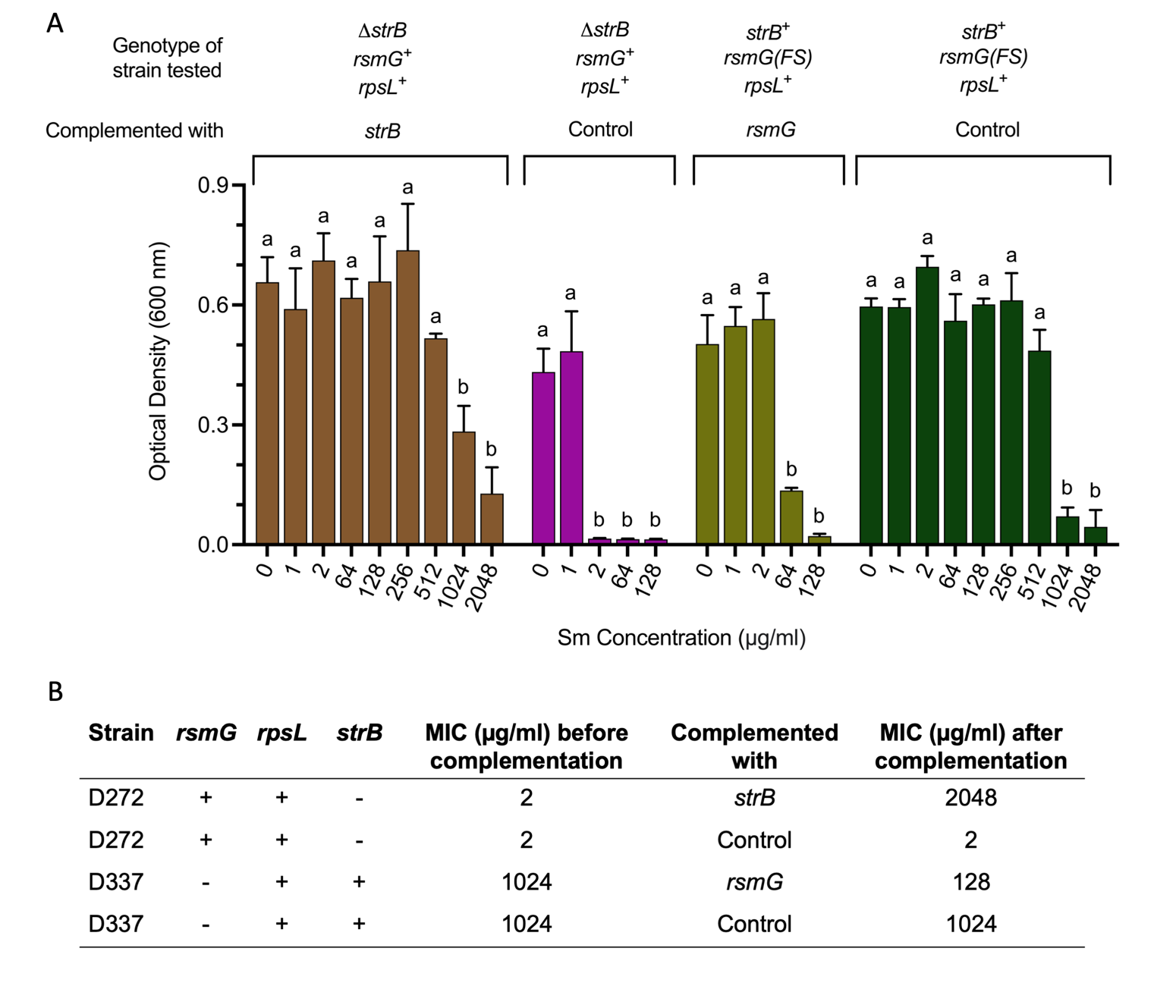
**

**FIG S1** Complementation with plasmid-borne *rsmG* and *strB* genes in *A. fabrum*. (A) Optical density measurements were taken 20 hours after inoculation of 200-µl cultures in 96-well plates. Genotype and complementation descriptions are given above each set of growth values. Error bars show standard deviation from the mean (n=3). Different letters denote statistically significant differences (P<0.05) according to a Tukey multiple comparison test. (B) Another representation of data shown in (A), indicating MIC values.
