# Supplementary material for: Three genes controlling streptomycin susceptibility in Agrobacterium fabrum
Source: J Bacteriol. 2023 Sep 11;205(9):e00165-23. doi: 10.1128/jb.00165-23 (PMC10521367; doi:10.1128/jb.00165-23)
Supplement: Supplemental material — Tables S1 to S5 and plasmid sequences. [file jb.00165-23-s0002.docx]

Supplemental Material for “Three genes controlling streptomycin susceptibility in *Agrobacterium fabrum*”

Table S1. Analysis of seven independently isolated *E. coli* streptomycin-resistant mutants that arose after selecting wild-type *E. coli* (MG1655) on Sm (200 µg/ml) for spontaneous mutations.

| Strain Name | Gene containing spontaneous mutation | DNA mutation | Protein change |
| --- | --- | --- | --- |
| SmR1 | *rpsL* | 129A>C | K43N |
| SmR2 | *rpsL* | 129A>C | K43N |
| SmR3 | *rpsL* | 263A>G | K89R |
| SmR4 | *rpsL* | 263A>G | K89R |
| SmR5 | *rpsL* | 128A>C | K43T |
| SmR6 | *rpsL* | 129A>C | K43N |
| SmR7 | *rpsL* | 128A>C | K43T |

Table S2. Transposon insertions of fifteen streptomycin-resistant mutants after transposon mutagenesis of *Agrobacterium fabrum* UBAPF2.

| Sample Name | Nucleotide Sequence After GAGACAG | Insertion Location | Gene Disrupted | Gene Directionality |
| --- | --- | --- | --- | --- |
| m32 01 | (28)TGGCGGCGCCGACCCGCTGTTTTCAGCTTGCGTTGCAGATC | 643,606 | *Atu3587* | + |
| m32 02 | (178)CCTTGCCGGTCGCCGCGATATCGAGCTGCTGTCCATTCCA | 1,234,994 | *speB* | - |
| m32 03 | (10)CGCGGGCTCGGCCAGCTTGATATTCGCGGCTCTGACGTG | 1,235,108 | *speB* | - |
| m32 04 | (22)TTCCGCAAGGCTGAGGCGGACGGCGGCATACTTGTCTGCC | 2,071,917 | *tnp* | + |
| m32 05 | (226)GCAGCGCGGCGATGCGCGAGGATTTGCTCAACAACGCCGAC | 1,234,989 | *speB* | - |
| m32 06 | (62)GCCGCAACCGACCCAACCCATGCGGGCGTTCCTCTCCTTC | 1,235,913 | IG(*speB*, *rpsI*) | -/- |
| m32 07 | (150)CCGAATACCCTCGCAACCCTTTGAAAAGGCAGGACAAGGAC | 1,235,069 | *speB* | - |
| m32 08 | (74)GATTCCGAATACCCTCGCAACCCTTTGAAAAGGCAGGACAA | 1,234,997 | *speB* | - |
| m32 09 | (194)AATTCGCTAACCGAGGCTTCTTGAGGCCACGGGTGA | 1,880,815 | *Atu4718* | + |
| m32 10 | (111)GCCCGTTTCGGTTATCAGTCGCGGTTCTTGTGCGGTGTCTGCCATCTGGCC | 94,413 | *Atu0090* | - |
| m32 11 | (87)CGCCTTGCCGGTCGCCGCGATATCGAGCTGCTGTCCATTC | 1,234,905 | IG(*argC*, *speB*) | -/- |
| m32 12 | (99)AACATAGAGTGCGTCGTCGCCGGCGGTATCGCGAACCAGTGTT | 293,679 | IG(*Atu0300*, *dnaN*) | -/- |
| m32 13 | (198)GCCGGGGCCACCGTTGCAATGTATATGCTTGGCCTGCGT | 1,235,212 | *speB* | - |
| m32 15 | (13)CATGATCGCGTCGTTGCTATCCACCAGCTTCAGATG | 777,498 | *Atu0781* | + |
| m32 16 | (58)ACGGTGAACACCGTGTGTAAAACAACCTGTAAAAT | 430,893 | IG(*Atu0435*, *Atu0436*) | +/+ |

Table S3. Bacterial strains used in this study.

| Strain Name | Purpose/Description | Antibiotic Resistance |
| --- | --- | --- |
| UBAPF2 | Plasmid-cured derivative of *Agrobacterium fabrum* (Also known as C237) | Rf |
| BB01 | SmR derivative of UBAPF2 (Also known as D233) | Rf, Sm |
| BL01 | SmR derivative of UBAPF2 (Also known as D234) | Rf, Sm |
| BM01 | SmR derivative of UBAPF2 (Also known as D231) | Rf, Sm |
| CI01 | SmR derivative of UBAPF2 (Also known as D229) | Rf, Sm |
| IW01 | SmR derivative of UBAPF2 (Also known as D232) | Rf, Sm |
| YS01 | SmR derivative of UBAPF2 (Also known as D230) | Rf, Sm |
| B001 | DH5ɑ strain harboring helper plasmid pRK600 | Cm |
| *strB*+ *rsmG(FS) rpsL*+ | Derivative of UBAPF2 with *rsmG* frameshift mutation 176^177insA (Also known as D337) | Rf, Sm |
| *strB+ rsmG+ rpsL(K43R)* | Derivative of UBAPF2 with *rpsL* mutation 128A>G resulting in the K43R variant (Also known as D338) | Rf, Sm |
| Δ*strB* *rsmG*+ *rpsL*+ | Δ*strB* derivative of UBAPF2 (Also known as D272) | Rf |
| Δ*strB* *rsmG(FS) rpsL*+ | Derivative of D272 with *rsmG* frameshift mutation 176^177insA (Also known as D339) | Rf |
| Δ*strB* *rsmG*+ *rpsL(K43R)* | Derivative of D272 with *rpsL* mutation 128A>G resulting in the K43R variant (Also known as D340) | Rf, Sm |

Table S4. Plasmids used in this study.

| Plasmid Name | Purpose/Description | Antibiotic Resistance |
| --- | --- | --- |
| pJG1108 | Parent plasmid for in-frame deletions (*p15Aori gus sacB kanR RK2oriT*) | Km |
| pJG1197 | pJG1108 derivative for deleting *strB* | Km |
| pJG1226 | Parent plasmid for constitutive expression of *strB* | Cm |
| pDC76 | pJG1226 derivative to express *strB* in *E. coli* | Cm |

Table S5. Primers used in this study.

| Primer Name | Sequence | Purpose/Description |
| --- | --- | --- |
| oDC103 | CGCTCTAGACTGTTTGCCGCCACGCT | Left homology region forward |
| oDC104 | TGCCCCGGGACCATCAAGGCAGTCCTGAAAC | Left homology region reverse |
| oDC105 | TGGCCCGGGGGCCGGAAAAGACGGCG | Right homology region forward |
| oDC106 | CGCGTCGACGGACCTTGCGGAAGGCAC | Right homology region reverse |
| oDC107 | TCGTCCATCGCCTGGGTG | *strB* forward |
| oDC108 | GGGCATGATCGTGCAGGT | *strB* reverse |
| oDC196 | cgcTCTAGAcgCTTAAGGCCTCCCAATC | Amplify pJG1226 forward |
| oDC197 | tagcctaagcTTAcgcAccAc | Amplify pJG1226 reverse |
| oDC198 | cgcAAGCTTTAGGAGGTATATATGATCAGCAATACGCCGTC | *strB* forward |
| oDC199 | cgcTCTAGAGTCGAAGTCAGGGACTGACC | *strB* reverse |
| Seq_rsmGStop | CCGCCTTTCTGGTTCGC | *rsmG* forward |
| Seq_rsmStart | CGCACTTCTGCGGAAAGGT | *rsmG* reverse |
| 2235 | CACGTTTTGCGCATGAGGAT | *rpsL* forward |
| 2236 | TCTGCACTATGGCGACGGGA | *rpsL* reverse |
| 2273 | Gcaaggcgacaaggtgctg | Sequence verification pDC76 forward |
| 2274 | CGGTTCAAAGAGTTGGTAGCTC | Sequence verification pDC76 reverse |
| 2133 | GTTCCGGCAAATATACTG | First-round arbitrary PCR forward |
| 2135 | CCACGCGTCGACTAGTACNNNNNNNNNNACGCC | First-round arbitrary PCR reverse |
| 2134 | GAAATAGGTGTTGACATTATTCC | Second-round arbitrary PCR forward |
| 2137 | CCACGCGTCGACTAGTAC | Second-round arbitrary PCR reverse |
| CD49 | GCCTACTTCACCTATCCTGCC | PCR check *strB* homology regions forward |
| CD50 | GGACCATGGTTATACCTCCTTC | PCR check *strB* homology regions reverse |
| For1 | ACAGCCGCGTTGTCGAGC | Sequence verification of *strB* region forward 1 |
| For2 | GCCCATATGCCGACCATC | Sequence verification of *strB* region forward 2 |
| For3 | CCTCGATCCCGCTTTCGC | Sequence verification of *strB* region forward 3 |
| For4 | TCCTCTGCCTGCCGGATG | Sequence verification of *strB* region forward 4 |
| For5 | CAGGGCATGATCGTGCAG | Sequence verification of *strB* region forward 5 |
| Rev1 | GTCAGTTCGTAAACGATGCC | Sequence verification of *strB* region *reverse 1* |

Plasmid Sequences

**pJG1108 (5544 bp)**

β-glucuronidase (*gus*): 79-1890 bp

Secreted levansucrase (*sacB*): 1910-3334 bp

Km resistance (*kanR*): 3513-4307 bp

p15A *ori*: 4587-5132 bp

RK2 *oriT*: 5361-5470 bp

TCTAGAccaGTCGACttacTTGACTtgtgagcggataacttcTATATGatgtggacacTTTAAGAAGGAGGTataaccATGGTCCGTCCTGTAGAAACCCCAACCCGTGAAATCAAAAAACTCGACGGCCTGTGGGCATTCAGTCTGGATCGCGAAAACTGTGGAATTGATCAGCGTTGGTGGGAAAGCGCGTTACAAGAAAGCCGGGCAATTGCTGTGCCAGGCAGTTTTAACGATCAGTTCGCCGATGCAGATATTCGTAATTATGCGGGCAACGTCTGGTATCAGCGCGAAGTCTTTATACCGAAAGGTTGGGCAGGCCAGCGTATCGTGCTGCGTTTCGATGCGGTCACTCATTACGGCAAAGTGTGGGTCAATAATCAGGAAGTGATGGAGCATCAGGGCGGCTATACGCCATTTGAAGCCGATGTCACGCCGTATGTTATTGCCGGGAAAAGTGTACGTATCACCGTTTGTGTGAACAACGAACTGAACTGGCAGACTATCCCGCCGGGAATGGTGATTACCGACGAAAACGGCAAGAAAAAGCAGTCTTACTTCCATGATTTCTTTAACTATGCCGGAATCCATCGCAGCGTAATGCTCTACACCACGCCGAACACCTGGGTGGACGATATCACCGTGGTGACGCATGTCGCGCAAGACTGTAACCACGCGTCTGTTGACTGGCAGGTGGTGGCCAATGGTGATGTCAGCGTTGAACTGCGTGATGCGGATCAACAGGTGGTTGCAACTGGACAAGGCACTAGCGGGACTTTGCAAGTGGTGAATCCGCACCTCTGGCAACCGGGTGAAGGTTATCTCTATGAACTGTGCGTCACAGCCAAAAGCCAGACAGAGTGTGATATCTACCCGCTTCGCGTCGGCATCCGGTCAGTGGCAGTGAAGGGCGAACAGTTCCTGATTAACCACAAACCGTTCTACTTTACTGGCTTTGGTCGTCATGAAGATGCGGACTTGCGTGGCAAAGGATTCGATAACGTGCTGATGGTGCACGACCACGCATTAATGGACTGGATTGGGGCCAACTCCTACCGTACCTCGCATTACCCTTACGCTGAAGAGATGCTCGACTGGGCAGATGAACATGGCATCGTGGTGATTGATGAAACTGCTGCTGTCGGCTTTAACCTCTCTTTAGGCATTGGTTTCGAAGCGGGCAACAAGCCGAAAGAACTGTACAGCGAAGAGGCAGTCAACGGGGAAACTCAGCAAGCGCACTTACAGGCGATTAAAGAGCTGATAGCGCGTGACAAAAACCACCCAAGCGTGGTGATGTGGAGTATTGCCAACGAACCGGATACCCGTCCGCAAGGTGCACGGGAATATTTCGCGCCACTGGCGGAAGCAACGCGTAAACTCGACCCGACGCGTCCGATCACCTGCGTCAATGTAATGTTCTGCGACGCTCACACCGATACCATCAGCGATCTCTTTGATGTGCTGTGCCTGAACCGTTATTACGGATGGTATGTCCAAAGCGGCGATTTGGAAACGGCAGAGAAGGTACTGGAAAAAGAACTTCTGGCCTGGCAGGAGAAACTGCATCAGCCGATTATCATCACCGAATACGGCGTGGATACGTTAGCCGGGCTGCACTCAATGTACACCGACATGTGGAGTGAAGAGTATCAGTGTGCATGGCTGGATATGTATCACCGCGTCTTTGATCGCGTCAGCGCCGTCGTCGGTGAACAGGTATGGAATTTCGCCGATTTTGCGACCTCGCAAGGCATATTGCGCGTTGGCGGTAACAAGAAAGGGATCTTCACTCGCGACCGCAAACCGAAGTCGGCGGCTTTTCTGCTGCAAAAACGCTGGACTGGCATGAACTTCGGTGAAAAACCGCAGCAGGGAGGCAAACAAtGAGCTCaaggagacatgaacgATGAACATCAAAAAGTTTGCAAAACAAGCAACAGTATTAACCTTTACTACCGCACTGCTGGCAGGAGGCGCAACTCAAGCGTTTGCGAAAGAAACGAACCAAAAGCCATATAAGGAAACATACGGCATTTCCCATATTACACGCCATGATATGCTGCAAATCCCTGAACAGCAAAAAAATGAAAAATATCAAGTTCCTGAATTCGATTCGTCCACAATTAAAAATATCTCTTCTGCAAAAGGCCTGGACGTTTGGGACAGCTGGCCATTACAAAACGCTGACGGCACTGTCGCAAACTATCGCGGCTACCACATCGTCTTTGCATTAGCCGGAGATCCTAAAAATGCGGATGACACATCGATTTACATGTTCTATCAAAAAGTCGGCGAAACTTCTATTGACAGCTGGAAAAACGCTGGCCGCGTCTTTAAAGACAGCGACAAATTCGATGCAAATGATTCTATCCTAAAAGACCAAACACAAGAATGGTCAGGTTCAGCCACATTTACATCTGACGGAAAAATCCGTTTATTCTACACTGATTTCTCCGGTAAACATTACGGCAAACAAACACTGACAACTGCACAAGTTAACGTATCAGCATCAGACAGCTCTTTGAACATCAACGGTGTAGAGGATTATAAATCAATCTTTGACGGTGACGGAAAAACGTATCAAAATGTACAGCAGTTCATCGATGAAGGCAACTACAGCTCAGGCGACAACCATACGCTGAGAGATCCTCACTACGTAGAAGATAAAGGCCACAAATACTTAGTATTTGAAGCAAACACTGGAACTGAAGATGGCTACCAAGGCGAAGAATCTTTATTTAACAAAGCATACTATGGCAAAAGCACATCATTCTTCCGTCAAGAAAGTCAAAAACTTCTGCAAAGCGATAAAAAACGCACGGCTGAGTTAGCAAACGGCGCTCTCGGTATGATTGAGCTAAACGATGATTACACACTGAAAAAAGTGATGAAACCGCTGATTGCATCTAACACAGTAACAGATGAAATTGAACGCGCGAACGTCTTTAAAATGAACGGCAAATGGTACCTGTTCACTGACTCCCGCGGATCAAAAATGACGATTGACGGCATTTCGTCTAACGATATTTACATGCTTGGTTATGTTTCTAATTCTTTAACTGGCCCATACAAGCCGCTGAACAAAACTGGCCTTGTGTTAAAAATGGATCTTGATCCTAACGATGTAACCTTTACTTACTCACACTTCGCTGTACCTCAAGCGAAAGGAAACAATGTCGTGGTGATTACAAGCTATATGACAAACAGAGGATTCTACGCAGACAAACAATCAACGTTTGCGCCAAGCTTCCTGCTGAACATCAAAGGCAAGAAAACATCTGTTGTCAAAGACAGCATCCTTGAACAAGGACAATTAACAGTTAACAAATAAaaacgcaaaagaaaatgccgatGGATCCacagcaagcgaaccggaattgccagctggggcgccctctggtaaggttgggaagccctgcaaagtaaactggatggctttcttgccgccaaggatctgatggcgcaggggatcaagatctgatcaagagacaggatgaggatcgtttcgcATGATTGAACAAGATGGATTGCACGCAGGTTCTCCGGCCGCTTGGGTGGAGAGGCTATTCGGCTATGACTGGGCACAACAGACAATCGGCTGCTCTGATGCCGCCGTGTTCCGGCTGTCAGCGCAGGGGCGCCCGGTTCTTTTTGTCAAGACCGACCTGTCCGGTGCCCTGAATGAACTGCAGGACGAGGCAGCGCGGCTATCGTGGCTGGCCACGACGGGCGTTCCTTGCGCAGCTGTGCTCGACGTTGTCACTGAAGCGGGAAGGGACTGGCTGCTATTGGGCGAAGTGCCGGGGCAGGATCTCCTGTCATCTCACCTTGCTCCTGCCGAGAAAGTATCCATCATGGCTGATGCAATGCGGCGGCTGCATACGCTTGATCCGGCTACCTGCCCATTCGACCACCAAGCGAAACATCGCATCGAGCGAGCACGTACTCGGATGGAAGCCGGTCTTGTCGATCAGGATGATCTGGACGAAGAGCATCAGGGGCTCGCGCCAGCCGAACTGTTCGCCAGGCTCAAGGCGCGCATGCCCGACGGCGAGGATCTCGTCGTGACCCATGGCGATGCCTGCTTGCCGAATATCATGGTGGAAAATGGCCGCTTTTCTGGATTCATCGACTGTGGCCGGCTGGGTGTGGCGGACCGCTATCAGGACATAGCGTTGGCTACCCGTGATATTGCTGAAGAGCTTGGCGGCGAATGGGCTGACCGCTTCCTCGTGCTTTACGGTATCGCCGCTCCCGATTCGCAGCGCATCGCCTTCTATCGCCTTCTTGACGAGTTCTTCTGAcccGGTACCtcagcgctagcggagtgtatactggcttactatgttggcactgatgagggtgtcagtgaagtgcttcatgtggcaggagaaaaaaggctgcaccggtgcgtcagcagaatatgtgatacaggatatattccgcttcctcgctcactgactcgctacgctcggtcgttcgactgcggcgagcggaaatggcttacgaacggggcggagatttcctggaagatgccaggaagatacttaacagggaagtgagagggccgcggcaaagccgtttttccataggctccgcccccctgacaagcatcacgaaatctgacgctcaaatcagtggtggcgaaacccgacaggactataaagataccaggcgtttccccctggcggctccctcgtgcgctctcctgttcctgcctttcggtttaccggtgtcattccgctgttatggccgcgtttgtctcattccacgcctgacactcagttccgggtaggcagttcgctccaagctggactgtatgcacgaaccccccgttcagtccgaccgctgcgccttatccggtaactatcgtcttgagtccaacccggaaagacatgcaaaagcaccactggcagcagccactggtaattgatttagaggagttagtcttgaagtcatgcgccggttaaggctaaactgaaaggacaagttttggtgactgcgctcctccaagccagttacctcggttcaaagagttggtagctcagagaaccttcgaaaaaccgccctgcaaggcggttttttcgttttcagagcaagagattacgcgcagaccaaaacgatctcaagaagatcatcttattaaggggtctgacgctcagtggaacgaaaactcacgttaagggattttggtcatgagattatcaaaaaggatcttcacctagatccttttaaattaaaaatgaagttttaaatcaatctaaagtatatatgagtaaacttggtctgacagttaccaatgcttaatcagactaGAGCTTCCATCCGCTTGCCCTCATCTGTTACGCCGGCGGTAGCCGGCCAGCCTCGCAGAGCAGGATTCCCGTTGAGCACCGCCAGGTGCGAATAAGGGACAGTGAAGAAGGAACACCCGCTCGCGGGTGGGCCTACTTCACCTATCCTGCCCGGCTGACGCCGTTGGATACACCAAGGAAAGTCTACACGAACCCTTTGGCAAAATCCTGTATATCGTGCGAATTG

**pJG1226 (2390 bp)**

Cm resistance (CmR): 87-746 bp

p15A ori: 860-1405 bp

gagctcTAGGGATAACAGGGTAATgcTCGACAattagtcatccggctcgTATAATgtgtggaagCTAGActttaggaggtatacatATGgagaaaaaaatcactggatataccaccgttgatatatcccaatggcatcgtaaagaacattttgaggcatttcagtcagttgctcaatgtacctataaccagaccgttcagctggatattacggcctttttaaagaccgtaaagaaaaataagcacaagttttatccggcctttattcacattcttgcccgcctgatgaatgctcatccggaattccgtatggcaatgaaagacggtgagctggtgatatgggatagtgttcacccttgttacaccgttttccatgagcaaactgaaacgttttcatcgctctggagtgaataccacgacgatttccggcagtttctacacatatattcgcaagatgtggcgtgttacggtgaaaacctggcctatttccctaaagggtttattgagaatatgtttttcgtctcagccaatccctgggtgagtttcaccagttttgatttaaacgtggccaatatggacaacttcttcgcccccgttttcaccatgggcaaatattatacgcaaggcgacaaggtgctgatgccgctggcgattcaggttcatcatgccgtTtgtgatggcttccatgtcggcagaatgcttaatgaattacaacagtactgcgatgagtggcagggTggTgcgTAAgcttaggctagctgacacgCTTAAGGCCTCCCAATCGGGAGGCCTTTTTTTgaccgATTACCCTGTTATCCCTAGTTCCACTGAGCGTCAGACCCCTTAATAAGATGATCTTCTTGAGATCGTTTTGGTCTGCGCGTAATCTCTTGCTCTGAAAACGAAAAAACCGCCTTGCAGGGCGGTTTTTCGAAGGTTCTCTGAGCTACCAACTCTTTGAACCGAGGTAACTGGCTTGGAGGAGCGCAGTCACCAAAACTTGTCCTTTCAGTTTAGCCTTAACCGGCGCATGACTTCAAGACTAACTCCTCTAAATCAATTACCAGTGGCTGCTGCCAGTGGTGCTTTTGCATGTCTTTCCGGGTTGGACTCAAGACGATAGTTACCGGATAAGGCGCAGCGGTCGGACTGAACGGGGGGTTCGTGCATACAGTCCAGCTTGGAGCGAACTGCCTACCCGGAACTGAGTGTCAGGCGTGGAATGAGACAAACGCGGCCATAACAGCGGAATGACACCGGTAAACCGAAAGGCAGGAACAGGAGAGCGCACGAGGGAGCCGCCAGGGGGAAACGCCTGGTATCTTTATAGTCCTGTCGGGTTTCGCCACCACTGATTTGAGCGTCAGATTTCGTGATGCTTGTCAGGGGGGCGGAGCCTATGGAAAAACGGCTTTGCCGCGGCCCTCTCACTTCCCTGTTAAGTATCTTCCTGGCATCTTCCAGGAAATCTCCGCCCCGTTCGTAAGCCATTTCCGCTCGCCGCAGTCGAACGACC
